# Minimally invasive cardiac surgery - A single centre experience

**DOI:** 10.1186/1749-8090-10-S1-A210

**Published:** 2015-12-16

**Authors:** Swapnil Deshpande, Purushottam Deshpande, Shrikrishna Deshpande, Mukund Deshpande, Irshad Ahmed, Jyoti Panhekar

**Affiliations:** 1 Dr. KG Deshpande Memorial Centre, Nagpur, Maharashtra, 440010, India

## Background/Introduction

Minimally Invasive Cardiac Surgery is the Future of Cardiac Surgery. The patients who undergo these surgeries have better post-operative recovery, better cosmesis and faster recovery times.

## Aims/Objectives

We present our data of 58 cases performed at our center in last 4 years.

## Method

18 mitral valve replacements, 20 ASD closures, 14, Off pump CABGs, 6 Aortic Valve Replacements.

Off the 14 CABGs, 3 were Hybrid Procedures.

## Results

Only one conversion to Median Sternotomy was necessary of the 58 cases which were operated. The operative time was definitely longer compared to the standard procedures but postoperative bleeding, ICU stay and Postoperative pain and ambulation were significantly smaller than standard surgeries. The patients have significantly reduced times to get back to complete work so the surgery also reduces the economic burden of the patients.

## Discussion/Conclusion

Minimally invasive cardiac surgery is here to stay in cardiac surgery practice. Proper patient selection is necessary at least in the initial stages to avoid surgical misadventures. The availability of HYBRID Cardiac surgery suites definitely reduces the peripheral vascular complications. With proper training and availability of resources minimally invasive cardiac surgery can be performed at most centres with acceptable results.

